# The Mayor-Elect

**Published:** 1897-12

**Authors:** 


A Monthly Review of Medicine and Surgery.

*EDITORS:*

THOMAS LOTHROP, M. D. - - WM. WARREN POTTER, M. D.

All communications, whether of a literary or business nature, should be addressed to the managing editor: 284 Franklin Street, Buffalo, N. Y.

Vol. XXXVII. DECEMBER, 1897. No. 5.

### THE MAYOR-ELECT.

DR. CONRAD DIEHL was chosen Mayor of Buffalo at the recent election by a majority so considerable as to speak strongly of his personal popularity. Dr. Diehl has been a successful practitioner of medicine in Buffalo for the past thirty years, is well known to the profession and has a large acquaintance amongst the general public. The writer is not of Dr. Diehl's political party nor did he vote for him at the last election; nevertheless, he desires to tender the good-will of the Journal to the incoming Mayor, who will take office January 1, 1898, and to say, that while its pages are denied to politics in its popular sense, yet it takes a keen interest in public affairs involving medical questions, and it will be only too ready to lend any aid in its power to Dr. Diehl in his administration wherein it touches questions of public and private health, applauding him when he acts justly and in accordance with modern scientific thought, offering adverse though friendly criticism if he shall do otherwise, but which latter necessity we do not anticipate.

It has been stated by many persons that a physician is not competent to hold public office except in the line of his profession. We desire to controvert this statement in a most emphatic manner. Doubtless there are physicians, as there are other citizens, unfitted for public place, because ability of this sort is a personal equation, but it is not denied to doctors anymore than it is to other professions or occupations. The fact is, that were a congress of physicians gathered from the four corners of the earth it would be found to possess more learning and ability, more liberalism and general knowledge, more administrative tact and more resources than any other group of men possible to assemble outside the ranks of medicine. There is no reason why a physician, trained to thought and action, should not make a good legislator, a good mayor, a good governor, or a good president. On the other hand, there is every reason why men of education, activity and training, such as most physicians are, should enter the ranks of politics whenever their fellow-citizens in considerable numbers or large groups invite them to do so. The English parliament is adorned by the names of Playfair and Lister, and the German reichstag by the immortal Virchow, the great liberal leader ; Jules Simon, too, was a life senator of France. But we digress from the main purpose of this article.

In addition to the ordinary duties performed in the executive chair, the mayor of Buffalo is ex-officio president of the board of health and of the board of police. He also appoints two members of the board of public works, besides numerous other officers who will serve the city during his administration. It will thus be observed that the mayor comes in direct touch with all that most immediately influences the health and prosperity of this great city. We place health first because without it there can be no prosperity. We fear business men are often inclined to overlook this important fact, whereas with a physician in the mayor's chair it will be ever foremost and be the governing principle in all his public acts, as it must be the controlling element in his private life. If this principle is overlooked in time of general good health, how quickly in the shadow of an epidemic does every one rush to the doctor for aid, comfort and protection. Let anyone who doubts question the business men of the South, asking them what effect the recent epidemic of yellow fever has had upon their business interests.

The water supply of a city demands the greatest circumspection with a view to its preservation, untainted and free from disease germs. It will be impossible for Dr. Diehl to furnish the city with *free* water, but as mayor it will be his duty to see that the board of public works and the health department, acting together, conduct into every household *pure* water in ample quantities.

Unnecessary noises in great cities are factors that contribute to the causation, prolongation or exaggeration of ill health. This journal has lifted its voice time and time again on this subject since July, 1893, when it began to point out editorially this evil, but so far to very little purpose. The noise nuisance and the smoke nuisance go hand in hand and should be absolutely suppressed in so far as they are both unnecessary. There are perforce necessary noises as there is some necessary smoke, but we need not differentiate —Dr. Diehl is a diagnostician.

The tenement house question properly comes before the mayor for solution. If the workingmen are to be kept healthful their homes must be kept clean, and frequent inspections must be made by competent persons. Whenever evils are reported they should be promptly corrected at whatever cost. An outlay in this direction is economy.

Clean streets are other important factors in the promotion and preservation of health. Our beautiful asphalted streets and avenues are kept reasonably clean in summer and autumn, but in winter sometimes they become a disgrace to modern civilisation.[1] Snow and ice are allowed to freeze in deep layers and to thaw out into grooves, holes and ruts, making traffic unsafe and almost impossible in places. The street railways throw the snow in heaps at the sides, which adds to the dilemma, for now almost every street of importance is traversed by tramcars. Here is the opportunity for the employment of idle men who are always clamorous for work, and whose employment becomes an important question in economics, since there is no surer way to prevent sickness than to employ the idle. Clean streets must not be limited to the business sections, but even on the outskirts of the city, esspecially in the workingmen's quarters, should receive attention, for it is impossible to secure clean homes without clean streets. A city is no cleaner than its dirtiest quarter.

The enforcement of school ordinances and careful school hygienic regulations becomes part of the mayor's duties, and no man in Buffalo should better understand these problems than Dr. Diehl, since he himself has been a school examiner for many years

The police inspection of saloons and of houses of prostitution will necessarily entail much careful thought on the part of the mayor, because as a member of the board of police he becomes directly responsible for the administration of that important department. Fortunately, in the person of Gen. William S. Bull, Buffalo possesses one of the best superintendents of police she ever had, and in him Mayor Diehl will find a coadjutor of force and decision, who will aid in protecting the health of the young by keeping them aloof from these two places of serious temptation.

---

1. It may be noted that last winter the main thoroughfares were kept in better condition than usual. Snow was more promptly removed from them.

The city's poor, the almshouse and the county hospital, always appeal to the better sentiments of a generous people and become objects of special interest to the mayor, and if he is also a physician he is all the better prepared to deal generously and honestly with these delicate subjects. We commend them to Dr. Diehl as of the greatest importance.

Finally, let us suggest to the incoming mayor a keynote to his success as a chief magistrate—namely, that he puts only the best men in office that he can secure to begin with, and that he keeps them in place only so long as they continue to remain the best. Fortunately, Mayor Diehl will find a well-organised health department and a splendidly administered police force to aid him in the discharge of his multifarious and irksome duties, and we trust that the end of his administration will witness a higher health-rate in Buffalo, even though now it stands at the head of the list of cities of its class.

